# Supplementary material for: The interactome of intact mitochondria by cross-linking mass spectrometry provides evidence for coexisting respiratory supercomplexes
Source: Mol Cell Proteomics. 2017 Dec 8;17(2):216–32. doi: 10.1074/mcp.RA117.000470 (PMC5795388; doi:10.1074/mcp.RA117.000470)
Supplement: Supplemental Data [file supp_RA117.000470_133922_0_supp_23978_fzffwf.zip › spectra_annotation/mito_DR_spectra_annotation/370-1-1-1-6-1.pdf]

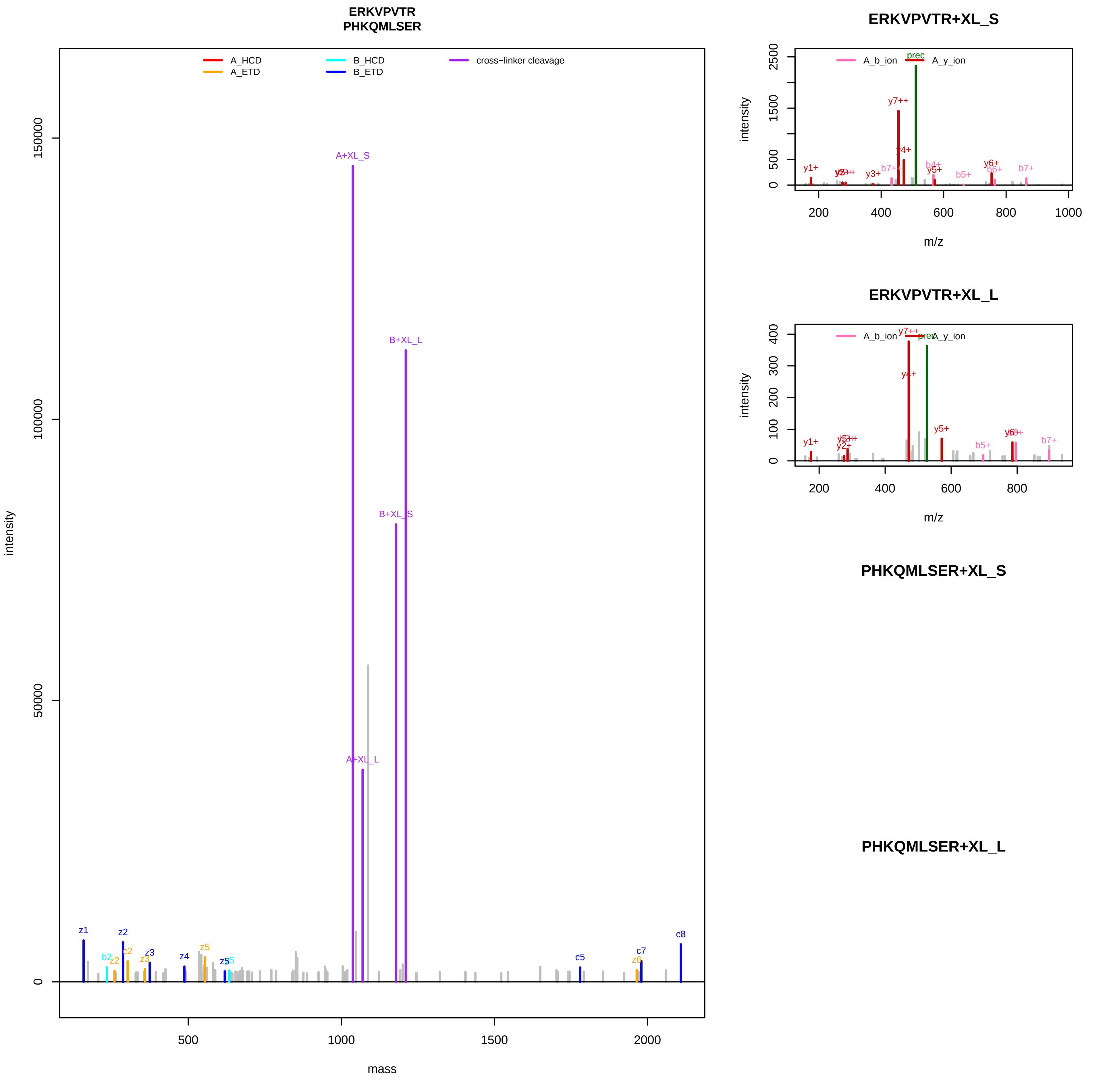

ERKVPVTR
PHKQMLSER
A_HCD
A_ETD
B_HCD
B_ETD
cross−linker cleavage
A+XL_S
B+XL_L
B+XL_S
A+XL_L
z1
z2
b2
c2
z3
z4
z5
c5
c7
c8
z6
intensity
mass
0
50000
100000
150000
500
1000
1500
2000
ERKVPVTR+XL_S
A_b_ion
A_y_ion
prec
y7++
y4+
y1+
y3+
b7++
b4+
y5+
b5+
y6+
b6+
b7+
m/z
ERKVPVTR+XL_L
y2+
PHKQMLSER+XL_S
PHKQMLSER+XL_L
